# The Impact of the Financial Crisis on Entrepreneurship Among Older Adults

**DOI:** 10.1093/geroni/igaa057.1491

**Published:** 2020-12-16

**Authors:** Julie Kim

**Affiliations:** University of California, Irvine, Irvine, California, United States

## Abstract

Older adults are assumed to be more averse to uncertainty than younger adults as economics and psychology studies based on prospect theory and related risk aversion theories show. Indeed, compared to younger adults, older adults engage in a smaller share of entrepreneurship. Yet adults 50 and over make up a steadily growing group of entrepreneurs in the US, and the 2008 financial crisis has not slowed them down. Sociological perspectives on older adults’ entrepreneurship consider disruption in the structural conditions of the labor market as a result of the economic crisis and a culture of active aging to argue that older adults’ entrepreneurial behaviors stem from structural and cultural roots. Analyses using 2003-2015 individual-level data from the Global Entrepreneurship Monitor show that an economic downturn did not thwart older adults’ entrepreneurial activities any more than it affected younger adults’ activities. Further, older adults’ odds of planning to start a business and actually starting a business were higher after the crisis than before the crisis. A closer examination reveals that older adults with less wealth generally have higher odds of pursuing entrepreneurship than other older adults. Meanwhile, wealthier older adults have higher odds of pursuing opportunity-driven entrepreneurship. Evidence suggests that the saliency of labor market conditions or the culture of active aging depends on older adults’ social location.

